# Extensive activation, tissue trafficking, turnover and functional impairment of NK cells in COVID-19 patients at disease onset associates with subsequent disease severity

**DOI:** 10.1371/journal.ppat.1009448

**Published:** 2021-04-16

**Authors:** Federica Bozzano, Chiara Dentone, Carola Perrone, Antonio Di Biagio, Daniela Fenoglio, Alessia Parodi, Malgorzata Mikulska, Bianca Bruzzone, Daniele Roberto Giacobbe, Antonio Vena, Lucia Taramasso, Laura Nicolini, Nicolò Patroniti, Paolo Pelosi, Angelo Gratarola, Raffaele De Palma, Gilberto Filaci, Matteo Bassetti, Andrea De Maria, on behalf of GECOVID study group

**Affiliations:** 1 Division of Infectious Diseases, Policlinico San Martino Hospital, Genoa, Italy; 2 Centre of Excellence for Biomedical Research and Department of Internal Medicine, University of Genoa, Genoa, Italy; 3 Department of Health Sciences, University of Genoa, Genoa, Italy; 4 Biotherapy Unit, Policlinico San Martino Hospital, Genoa, Italy; 5 Hygiene Unit, Policlinico San Martino Hospital, Genoa, Italy; 6 Anesthesia and Intensive Care, Policlinico San Martino Hospital, IRCCS for Oncology and Neurosciences, Genoa, Italy; 7 Department of Surgical Sciences and Integrated Diagnostics (DISC), University of Genoa, Genoa, Italy; 8 Internal Medicine Unit, Clinical Immunology and Translational Medicine, Policlinico San Martino Hospital, Genoa, Italy; 9 Department of Internal Medicine (DIMI), University of Genoa, Italy; The Peter Doherty Institute and Melbourne University, AUSTRALIA

## Abstract

The SARS-CoV-2 infection causes severe respiratory involvement (COVID-19) in 5–20% of patients through initial immune derangement, followed by intense cytokine production and vascular leakage. Evidence of immune involvement point to the participation of T, B, and NK cells in the lack of control of virus replication leading to COVID-19. NK cells contribute to early phases of virus control and to the regulation of adaptive responses. The precise mechanism of NK cell dysregulation is poorly understood, with little information on tissue margination or turnover. We investigated these aspects by multiparameter flow cytometry in a cohort of 28 patients hospitalized with early COVID-19.

Relevant decreases in CD56^bright^CD16+/- NK subsets were detected, with a shift of circulating NK cells toward more mature CD56^dim^CD16+KIR+NKG2A+ and “memory” KIR+CD57+CD85j+ cells with increased inhibitory NKG2A and KIR molecules. Impaired cytotoxicity and IFN-γ production were associated with conserved expression of natural cytotoxicity receptors and perforin. Moreover, intense NK cell activation with increased HLA-DR and CD69 expression was associated with the circulation of CD69+CD103+ CXCR6+ tissue-resident NK cells and of CD34+DNAM-1^bright^CXCR4+ inflammatory precursors to mature functional NK cells. Severe disease trajectories were directly associated with the proportion of CD34+DNAM-1^bright^CXCR4+ precursors and inversely associated with the proportion of NKG2D+ and of CD103+ NK cells.

Intense NK cell activation and trafficking to and from tissues occurs early in COVID-19, and is associated with subsequent disease progression, providing an insight into the mechanism of clinical deterioration. Strategies to positively manipulate tissue-resident NK cell responses may provide advantages to future therapeutic and vaccine approaches.

## Author summary

This is a detailed study of activating and inhibitory receptors in NK cells of COVID-19 patients when first admitted to the hospital for respiratory insufficiency. NK cells are known to be the first line of defense against invading viruses, and regulate downstream B and T cell responses, including antibody production. We observed intense NK cell activation with decreased functional activity, as well as intense circulation of putative tissue resident CD69+CD103+CXCR6+ NK cells, with a related surge in inflammatory CD34+ precursors from the bone marrow. The findings suggest that there is unprecedented trafficking of NK cells from peripheral tissues, their increased death with recruitment of emergency precursors from the bone marrow, and a relationship with the subsequent course of the disease of the patients. This in turn suggests possible areas of treatment and prevention.

## Introduction

The new strain of the large betacoronavirus family (severe acute respiratory syndrome coronavirus 2, or SARS-CoV-2) that is spreading as a global pathogen causing coronavirus-19 disease (COVID-19) [1, 2]] has caused an ongoing global pandemic with over 23 million infections (Worldometers [http://www.worldometers.info] The Real Time Statistics Project) [3].

SARS-CoV-2 is the seventh known strain of enveloped positive-strand RNA coronaviruses, which causes a range of diseases in humans [4], ranging from asymptomatic or mild non-respiratory disease in 80–90% of cases [5–7] to a severe disease requiring hospitalization and intensive oxygen support in 10–20% of cases.

The severity and mortality of COVID-19 is increased by age and by many comorbidities, including diabetes, obesity, and cardiovascular and pulmonary disease [8, 9]. It is, however, still largely unclear whether or to what extent disease severity is associated with virus replication and with derangements in the host response. There is an urgent need to focus on the immune dysregulation underlying early COVID-19 [10].

NK cells help clear virus-infected cells through multiple mechanisms, including direct contact, cytokine or chemokine secretion, and indirectly influencing lateral and downstream adaptive immune responses via their crosstalk with dendritic cells and T cells [11–13]. They are markedly activated during ongoing viral infection [14, 15] and contribute to viral control [16, 17], for example by memory-like responses [12], both directly and by regulating dendritic cell maturation and adaptive responses [11, 12]. Their derangement may thus be deranging not only direct virus control, but also the efficient organization of downstream T and B cell adaptive responses.

Profiling of innate immune responses to SARS-CoV-2 so has far shown that during COVID-19, there is a significant decrease in total peripheral blood lymphocytes of T and natural killer cells, which is associated with disease severity [18, 19]. The features of immune response dysregulation include unusually high cytokine plasma concentrations (TNFa, IL-6, IL-8, IL-10) and decreased T regulatory cells, with apparently unchanged T cell and NK production of IFN-gamma [20]. More recently, multiple derangements have been reported in COVID-19 patients, including T cell activation and oligoclonal plasmablast expansion with some Fc receptor dysregulation in innate cells (NK cells, monocytes) [21]. None of the immune parameters studied, however, correlate with disease trajectories beyond neutrophil to lymphocyte ratio [21]. Additional antiviral dysregulation has also been associated with increased expression of the inhibitory HLA-E-specific NKG2A+ receptor on T cells and NK cells that may decrease their antiviral function [22].

Overall, there has been limited research on the extent and type of NK cell derangement during SARS-CoV-2 infection, particularly with regard to the expression of the full array of the cells’ activating receptors (e.g., natural cytotoxicity receptors, NKG2D, DNAM-1, etc.), inhibitory receptors (e.g., NKG2A/CD94, CD85j, killer immunoglobulin-like receptors, KIRs), and circulating subsets, including CD56^dim^CD16+ effectors, less developmentally advanced CD56^bright^CD16+/- regulatory NK cells [23, 24], and CD56-CD16+ “exhausted” NK cells with dysfunctional properties [25, 26]. In addition, although decreases in peripheral NK cells are consistently associated with severe SARS-CoV-2 infection [19, 22], it is unclear whether this is due to NK cell trafficking to infected tissues, to cell leakage from involved tissues, or to cell death and turnover. It is also unclear whether or to what extent increased inhibitory signaling may favor virus replication and pathology.

In order to contribute to the ongoing work on innate immune landscape profiling, and to support immune intervention strategies, we have performed an in-depth analysis of NK cells, focusing on their peripheral distribution, function, trafficking, and turnover in hospitalized COVID-19 patients with different disease trajectories. The results indicate that an intense derangement of NK cell trafficking, activation, function, and turnover occurs early on, and is associated with the subsequent disease trajectory in hospitalized patients.

## Materials and methods

### Ethics statement

The study was carried out in accordance with the principles of the Declaration of Helsinki, and was approved by the Ethic Committee of the Liguria Region (N. CER Liguria 114/2020—ID 10420).

### Patients

The first 28 consecutive SARS-CoV-2 infected patients admitted with COVID-19 were enrolled to the study. Peripheral blood samples of 18 healthy uninfected donors (HD) were obtained from the blood bank (G. Gaslini Institute, Genoa, Italy, and IRCCS San Martino IST). All enrolled patients and subjects provided verbal informed consent.

This cohort was part of a larger coordinated effort of enrolling patients admitted to the infectious diseases division and collaborating units of our institute and to the COVID-19 ICU between March and June 2020. Sequential patients admitted to the Institute were enrolled at admission; none were lost to follow up. Sampling occurred after a mean±S.D. of 4.3±3.06 days from admission and after a 8.3±3.03 days from onset of symptoms. All the disease trajectories after admission and sampling were directly monitored, and the full disease course until either discharge or death was recorded. Clinical data were kept on record and used as needed. Clinical data from the whole cohort are reported elsewhere [27]. A cohort of HIV-1 infected patients, virologically suppressed for at least 18 months on successful antiretroviral treatment was included as control for inflammatory precursor analysis.

Disease severity was defined based on the need for oxygen support during the whole hospital stay until discharge. According to a modified WHO clinical progression scale [28], patients were grouped according to their need for inspiratory oxygen fraction support, as judged by the clinical team physicians, into the following categories: Grade 1, from 24% to ≤60% (WHO 4–5); Grade 2, 60% to non-invasive ventilation (WHO 6); Grade 3, mechanical ventilation requirements (WHO 7–9).

### Samples and cell culture

Peripheral blood (PB) (15 mL) was drawn by venipuncture of a peripheral vein within 72 hours from patient admittance to the unit, and after providing informed consent. Peripheral blood mononuclear cells (PBMCs) were obtained by density gradient centrifugation (Ficoll-Hypaque) and cryopreserved at –86°C and liquid nitrogen until processed.

### Antibodies

Commercial mouse anti-human mAbs are listed in the supplementary information. Anti-KIR2DL2/L3/S2 (CD158b1/b2,j), anti-KIR3DL1/S1 (CD158e1/e2), anti-KIR2DL1/S1 (CD158a/h), anti-NKG2A (Z270, IgG1; Z199, IgG2a), anti-CD85j (F278, IgG1) were kindly provided by Dr. D. Pende. All were produced in the laboratory (A.Moretta, Genova). Anti HLA-DR (D1-12, IgG2a) was kindly provided by Dr. R.S. Accolla (University of Insubria, Varese, Italy).

### Immunofluorescence analysis

Cells were analyzed by multicolor flow cytometry, as described previously[29]. For analysis, cells were gated using forward and side light scatter parameters (FACS Fortessa, BD, Mountain View, CA, USA, 10,000 events). Mean fluorescence intensity ratios (MFIr) were calculated using the formula MFI sample/MFI negative control, and express mean cell molecule density. Data were analyzed using FlowJo (Tree Star, Inc.) and FCS Express (DeNovo Software).

### Multidimensional data reduction analysis

Flow cytometric data were exported with compensated parameters in FCS Express software v6.03.0011 (DeNovo Software). A t-dependent stochastic neighbor embedding (t-SNE) analysis was performed on the transformed data for FSC-A, SSC-A, CD49, CD69, and CD103 markers using Barnes-Hut SNE (bh-SNE) approximations. This generated 2-D plots that clustered cells based on marker expression profiles.

### CD107a degranulation assay

PBMC were cocultured with target cell lines at a 1:1 E:T ratio in complete medium. For reverse-ADCC, cells were challenged using FcγR+ P815 target cells at 1:1 E:T ratio in complete medium in the absence or presence of PMA+Ionomycin. Anti-CD107a mAb (BD PharMingen) was added, as previously described [29]. Cells were surface-stained using anti-CD3FITC and anti-CD56PeCy7 mAbs.

### Statistical analysis

Statistical analysis was performed using the Mann-Whitney U tests for unpaired datasets for comparisons. Tests were two-sided. Analysis was performed using JMP 10.0 (SAS) unless stated otherwise.

## Results

### Study design, clinical cohort, and subset analysis of peripheral NK cells in hospitalized COVID-19 patients at the onset of the disease

Clinical and demographic data of the 28 patients are reported in Table 1. In line with previous reports[20, 22], we observed lower lymphocyte numbers and increasing ages for patients with overall higher severity of disease (Table 1).

**Table 1:** Demographic, laboratory and severity characteristics of the study cohort.

Severity Grade	All	Grade 1	Grade 2	Grade 3
**Patients (n°)**	28	6	11	11
**Age (yrs)**	72.10±13.09	76.83±7.33	75.18±8.81	68.9±16.02
**Male**	16	3	7	6
**Female**	12	3	4	5
**Temperature (°C)**	37.86±0.94	37.3±0.14	37.86±1.08	37.54±0.97
**Lymphocytes 10**^**9**^**/L**	0.89±0.52	1.08±0.6	0.92±0.25	0.79±0.44
**Monocytes 10**^**9**^**/L**	0.47±0.28	0.53±0.11	0.48±0.25	0.44±0.23
PCR mg/L *,#	107.35±90.8	5.65±3.88	102.4±67.69	137.56±96.59
**Fibrinogen g/L §.°**	5.53±3.07	3.69±0.54	5.81±1.81	5.55±2.03
**IL6 pg/ml**	191.56±239.16	6.8±33.7	262.41±447.86	202.57±225.13
**D-dimer ug/L**	4249.69±7324.05	1110.45±1082.65	7119.33±11594.95	1367.79±1005.73
**Ferritin μg/L**	1026.5±595.16	584±446	968.85±372.9	1116.37±847.99
**Intensive care unit**	8	0	0	11
**Exitus**	6	0	1	5

• p = 0.04 Wilcoxon all-group comparison; # p = 0.05 Grade 1 vs. Grade 2 (U-Test)

• p = 0.05 Wilcoxon all-group comparison; ° p = 0.03 Grade 1 vs. Grade 2 (U-Test)

**Fig 1:**
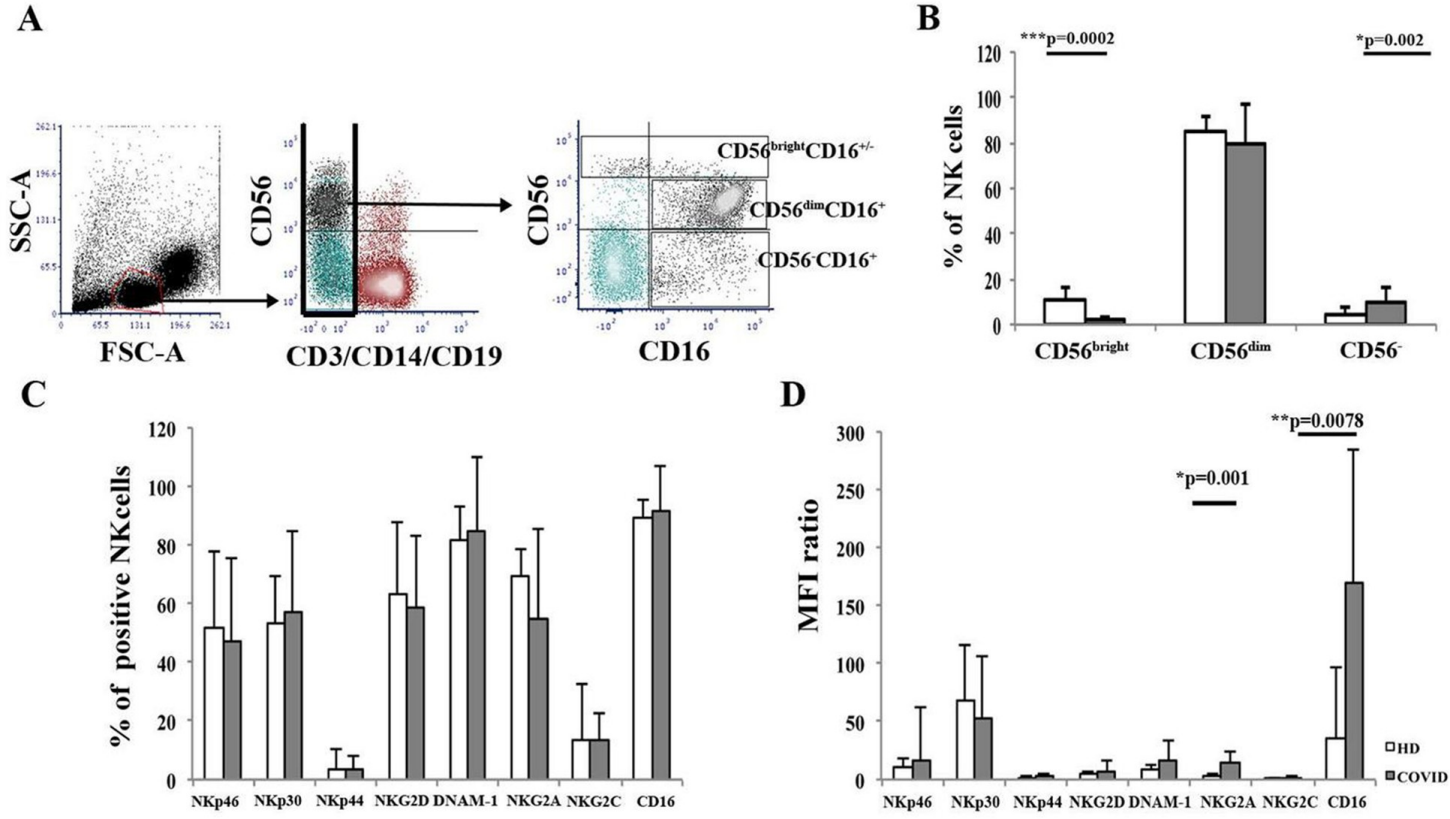
Flow cytometric analysis of peripheral blood NK cell subsets and receptors in COVID-19 patients. **Panel A:** Flow cytometric gating strategy. Following physical selection (FCS, SSC), CD3-CD19-CD14- cells are gated and further analyzed as NK cells expressing CD16 and CD56. Analysis of the acquisition of 10000 events. Log scale. **Panel B:** Analysis of peripheral blood NK cell subsets defined by CD16 and CD56 expression in COVID-19 patients (#28, grey columns) and HD (#18, white columns). CD56^bright^CD16+/- cells are indicated as CD56^bright^, CD56^dim^CD16+ NK cells are indicated as CD56^dim^; CD56-CD16+ NK cells are indicated as CD56^-^. Histograms show mean±SD. Cells are gated on CD3-CD19-CD14- cells. Significance by Mann-Whitney U-test analysis is indicated. The ratio between CD56^bright^CD16+/- and CD56^dim^CD16+ cells is indicated as CD56^bright^/CD56^dim^. Open histograms: HD. Greyed histogram: COVID-19. **Panel C:** Analysis of PB NK cell receptor expression. Histograms indicate the proportion of NK cells expressing each surface molecule. Cells are gated on CD3-CD19-CD14- cells. Significance by Mann-Whitney U-test analysis is indicated. Open histograms: HD. Greyed histogram: COVID-19. **Panel D:** Molecule density expression of NK cell molecules on PB NK cells. Molecule density is expressed as the MFI ratio. Histograms show mean±SD. Mann-Whitney U-test analysis is indicated.

Flow cytometric subset analysis of patients’ PB NK cells were compared to HD. The gating strategy and a dot plot representation are shown Fig 1A. Subset analysis according to CD56 and CD16 expression of CD3-CD14-CD19-cells showed a significant fourfold reduction of CD16+/-CD56^bright^cells, with a consequent remarkable drop in the ratio between CD16+/- CD56^bright^ and CD16+CD56^dim^ cells (Fig 1B; p<0.0003, U-test). A concomitant increase in CD16+CD56- “exhausted” NK cells (Fig 1B) was observed.

Comparable expression of NKp46, NKp30, NKp44, NKG2D, DNAM-1, and NKG2C activating receptors was detected on NK cells from patients and HD (Fig 1C and 1D). On the other hand, a significant increase of the inhibitory HLA-E-specific NKG2A molecule per-cell density was observed (17.27±8.98 vs. 3.62±1.39; p = 0.001, U-test), without changes in the frequency of NKG2A+ NK cells (52.7 ±26.6 vs. 69.63 ±8.73) (Fig 1C and 1D).

**Fig 2:**
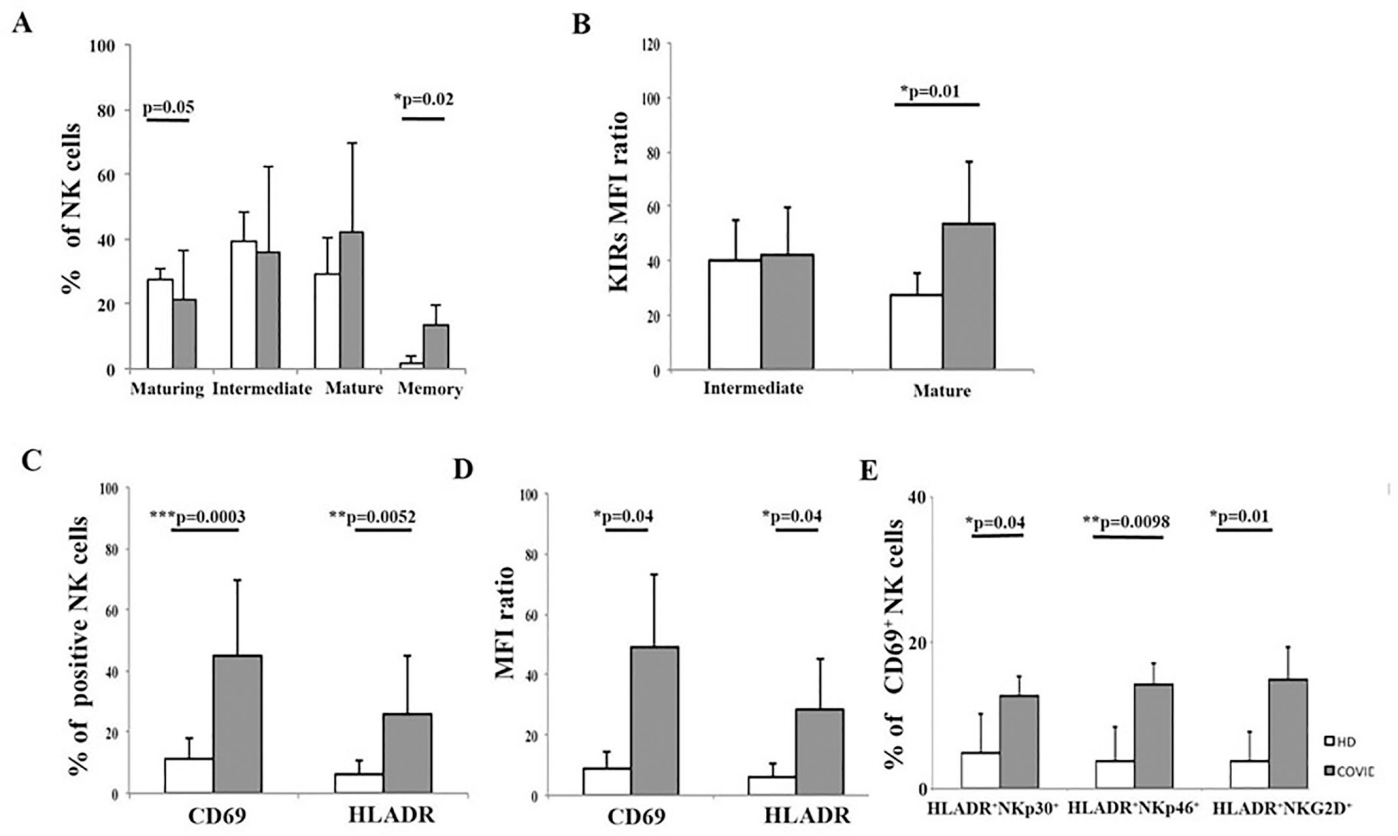
Flow cytometric analysis of peripheral blood NK cell development and activation in COVID-19 patients. Analysis is performed on CD3-CD14-CD19- cells by flow cytometry in COVID-19 patients (#28) and HD (#18). Histograms show mean±SD. Significance by Mann-Whitney U-test analysis is indicated. Open histograms: HD. Greyed histogram: COVID-19. **Panel A:** Analysis of circulating NK cell development according to NKG2A and KIR expression of PB cells. KIR and NKG2A were analyzed on CD3-CD14-CD19-CD56+CD16+ cells. Maturing = KIR-NKG2A+. Intermediate = KIR+NKG2A+. Mature = KIR+NKG2A-. Memory = CD85j+KIR+ CD57+NKG2A-. **Panel B:** MFI ratios of KIR expression in NK cells. MFIr = mean fluorescence intensity ratio calculated as (MFI sample–MFI neg control)/MFI neg control. MFIr expresses NK cell molecule density. **Panel C:** Proportions of NK cells expressing CD69 and HLA-DR. **Panel D:** MFI ratios of CD69 and HLA-DR on PB NK cells. MFIr = mean fluorescence intensity ratio calculated as (MFI sample–MFI neg control)/MFI neg control. MFIr expresses NK cell molecule density. MFIr expresses NK cell molecule density. **Panel E:** analysis of the expression of HLA-DR in NKp46+, NKp30+, or NKG2D+ NK cells.

Peripheral NK cell developmental status was then studied using the expression of NKG2A and KIR. A decrease in regulatory NKG2A+KIR- circulating NK cells was detected in patients, compared to HD, consistent with the decreased proportion of developmentally upstream CD16+/- CD56^bright^ cells (Fig 2A).

Taken together, these findings suggest an in vivo shift of NK cell PB subsets toward more mature effector stages during early COVID-19 symptomatic disease conditions.

### Circulating NK cells in COVID-19 are activated and enriched in tissue-resident cells

The observed shift in NK cell subsets with decreased CD16+/-CD56^bright^ and increased CD16+CD56- “exhausted” subsets raised the possibility of their accelerated peripheral development/maturation following their activation. Since overt systemic virus replication induces HLA-DR and CD69 expression on NK cells [15], we next studied their expression, and observed an increase in both HLA-DR+ and CD69+ NK cell proportions (Fig 2B), together with an increased HLA-DR molecule density (Fig 2C). The increase in the expression of HLA-DR was detected on all NK cells, independent of NCR expression, suggesting diffuse homogeneous NK cell activation (Fig 2D).

**Fig 3:**
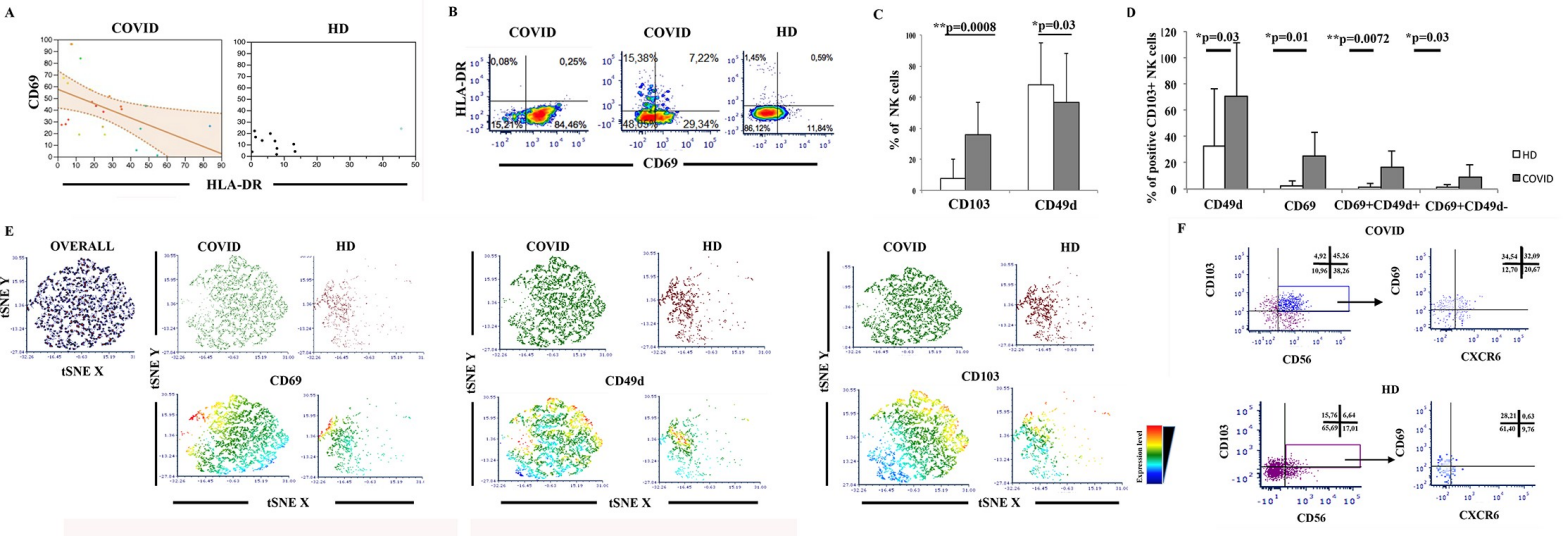
Flow cytometric and correlation analysis of the expression of activation and tissue-residency molecules on circulating NK cells in COVID-19 patients. **Panel A:** Correlation analysis of the expression of CD69 and of HLA-DR on PB CD56+CD16+ NK cells according to least squares linear regression. Slope and 95% confidence limits are plotted. p = 0.01. **Panel B:** Flow cytometric dot plot analysis of the expression of CD69 and of HLA-DR on circulating NK cells in different patients. CD69 and HLADR are mostly not coexpressed in COVID-19 patients. A representative HD profile is also shown. **Panel C:** Analysis of CD103 and of CD49d expression on PB NK cells in COVID-19 patients and HD. Histograms show mean±SD. Significant differences are indicated according to the Mann-Whitney U-test. **Panel D:** Increased coexpression of CD49d and CD69 in circulating CD103+ NK cells in COVID-19 patients. Histograms show mean±SD. Mann-Whitney U-test analysis is shown. **Panel E:** A tSNE plot showing distinct expression of CD69, CD49d, and CD103 in COVID-19 and HD. **Panel E:** A t-SNE representation of the coexpression of CD69, CD103, CD49d in PB NK cells. **Panel F:** Analysis of CXCR6 expression on PB NK cells in a representative sample of COVID-19 patients and HD. Dot plots show CD103 expression on CD3-CD14-CD19- NK cells and their coexpression of CD69 and/or CXCR6. The proportion of positive cells is indicated. COVID-19 patients have relevant circulation of CXCR6+CD69+CD103+NK cells, while HD have no such circulation (here 32.09% vs. 0.63%).

The expression of CD69 on NK cells was initially interpreted as a triggering molecule [30] associated with early activation events in vitro [31, 32]. The view on its function has recently been updated to include the definition of tissue residency [33–35]. Interestingly, the analysis of the relationship between CD69 and HLA-DR in COVID-19 patients showed that the frequency of PB NK cells expressing both surface antigens is increased in NK cells from COVID-19 patients with parallel increased molecule densities (Fig 2D and 2E). Unexpectedly, CD69 expression was inversely correlated to HLA-DR (p = 0.03, Spearman r = 0.24; Fig 3A). It was also occasionally observed in representative cytometric profiles (Fig 3B), and was not observed in HD.

In view of the role of CD69 in tissue-residency programs of NK cells, this finding could be related to increased trafficking of NK cells in and out of inflamed tissues. To verify this possibility, we analyzed the expression of integrin alpha E (CD103, the receptor for E-cadherin), which is thought to be a marker of tissue residency, and of alpha 4 integrin (CD49d, VLA-4, the receptor for VCAM and fibronectin), which is thought to be a marker of homing and extravasation to inflammation areas. Increased proportions of circulating NK cells expressing CD103 (p = 0.0034, U-test), with reduced CD49d+NK cells (p = 0.03. U-test; Fig 3C) were detected. Subset analysis on circulating CD103+CD56+ NK cells further showed that COVID-19 patients had increased proportions of CD49d+CD103+, CD69+CD103+, and CD69+CD49d+CD103+ NK cells (Fig 3D). Application of multidimensional data reduction analysis through t-dependent stochastic neighbor embedding (t-SNE) analysis confirmed discrete co-expression of CD49d, CD69, and CD103 in PB NK cells (Fig 3E). In addition, the observation that about 50% of CD103+ NK cells and one-third of circulating CD103+CD69+ NK cells coexpress CXCR6, which is a chemokine receptor associated to tissue residency, (Fig 3F), confirms that there is a panel of antigen expression in COVID-19 patients that is in line with a significant and unusual circulation and trafficking of tissue-resident NK cells.

### Cytotoxic NK cell function in COVID-19 patients is decreased upon triggering signals

**Fig 4:**
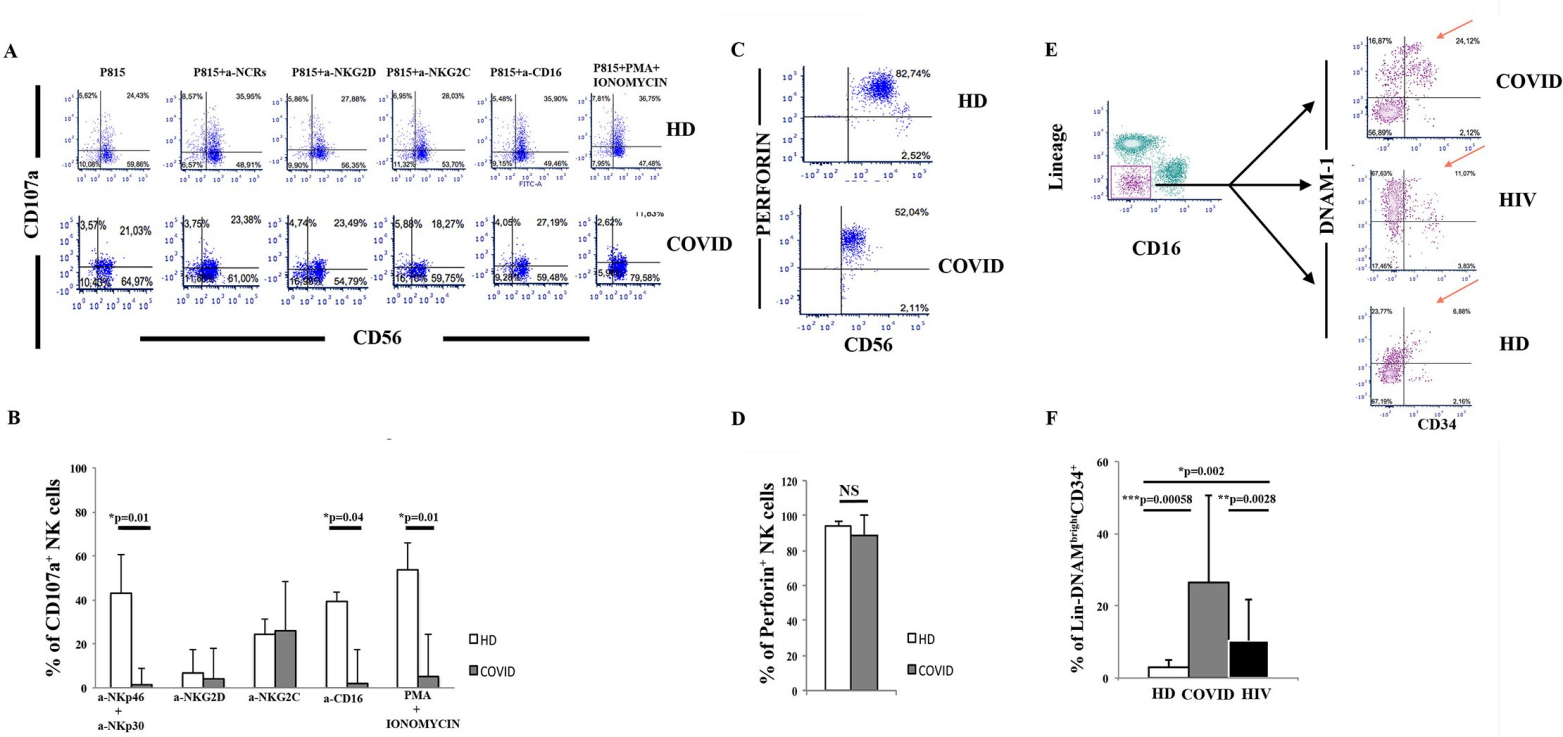
Functional study of circulating NK cells and of their peripheral turnover through analysis of “inflammatory” CD34+DNAM^bright^CXCR4+ precursors. **Panel A:** In vitro NK cell cytotoxicity assay by flow cytometric analysis of CD107a expression in a representative COVID-19 patient and HD. A redirected killing (reverse ADCC) is shown, using NKp46 and NKp30 NKG2D, NKG2C, CD16 mAb-mediated triggering in the presence of Fcγ^+^ P815 cells. Left: no mAb. Center: NCRs (α-NKp46+αNKp30, α- NKG2D, α-NKG2C, α-CD16). Right: the total lytic potential (as tested by maximal stimulation with PMA+ionomycin) of circulating NK cells is decreased in COVID-19 patients. Dots indicate CD107a degranulation 4 hours after NK cell triggering with the indicated stimulus. **Panel B:** In vitro cytotoxicity of PB NK cells as determined by CD107a expression in COVID-19 patients (n = 8) vs. HD (n = 8) is not inducible. Histograms show mean±SD. Mann-Whitney U-test analysis is shown. **Panel C:** Perforin expression by NK cells in COVID-19 patients is conserved. Representative flow cytometric analysis of CD3-CD19-CD14-CD56+ NK cells expressing perforin. 10000 events are analyzed. **Panel D:** The proportion of perforin+ NK cells is conserved in COVID-19 patients. Histograms indicate the proportion of Perf+ NK cells over total CD56+ NK cells, mean±SD. **Panel E:** Flow cytometric gating strategy and analysis of DNAM-1 and CD34 expression on Lin- CD16- cells in a representative COVID-19 and a HIV-1 patient and a control HD. Lineage = CD3, CD14, CD19, CD56. A representative COVID-19 patient (upper plot), HIV patient (middle plot), and HD (bottom plot) are shown. Box and arrow indicate the area of DNAM-1^bright^ appearance of CD34+ circulating precursors. **Panel F:** Cumulative circulation of CD34+DNAM-1^bright^CXCR4+ common lymphocyte precursors in COVID-19 patients (#28) is increased compared to HD (#18) and also to HIV-1 patients (#15). Histograms show mean±SD. Mann-Whitney U-test analysis is shown.

To study the cytotoxic function of NK cells mediated by the triggering of activating receptors and their overall cytotoxic potential, we performed a reverse ADCC assay investigating their CD107a degranulation, which is a direct correlate of cytotoxic activity [36]. Spontaneously increased cytotoxicity of freshly drawn unstimulated NK cells was observed in COVID-19 patients, but not in HD donors, as evaluated by CD107a production/expression (45.3±8.29% vs. 19.9%±11.45, p<0.01, U-test; Fig 4A). Redirected killing of FcγR+ P815 cells in the presence of anti-NKp30 and anti-NKp46 mAbs showed a reduced degranulation of NK cells in COVID-19 patients (42.95±18.69% vs. 0.31 ±8.53%; p<0.01, U-test; Fig 4A and 4B). A similar decrease in cytotoxicity was observed using anti-CD16 mAbs (39.06%±4.55 vs.1.94%±15.15% p = 0.04 U-test, Fig 4B) while a non-significant decrease was observed for NKG2C or NKG2D, In addition, a decreased total degranulation potential, as determined by maximal stimulation with PMA+ionomycin, was detected in COVID-19 patients (55.4 ±13,90% vs. 12.24 ±12.85%, p<0.01, U-test; Fig 4A and 4B) with conserved perforin contents comparable to HD (Fig 4C and 4D).

Thus, activated circulating NK cells expressing normal levels of triggering receptors have a significantly limited ability to induce additional cytotoxicity upon specific triggering, which is independent of defects in perforin or triggering receptor expression.

### Increased circulation of inflammatory CD34+DNAM-1^bright^CXCR4+ common lymphocyte precursors

The observation of relevant activation and tissue-trafficking of NK cells suggested a possible increased peripheral NK cell turnover: increased NK cell turnover is associated with the release of “inflammatory” Lin-CD34+DNAM-1^bright^CXCR4+ NK cell precursors from the Bone marrow(BM) that are detected in PB [29]]. They are conventionally identified as Lin-DNAM-1^bright^ cells by flow cytometric analysis, and generate mature KIR+ NK cell progenies [29].

To address NK cell turnover, as opposed to anergy, in view of the functional results, we studied the presence and frequency of Lin-CD34+DNAM-1^bright^CXCR4+ cells in COVID-19 patients, and compared them to HIV patients, who are known to have increased frequencies [29], as well as to HD, as a negative control group.

Flow cytometric dot plot analysis showed that the frequency of Lin-CD34+DNAM-1^bright^CXCR4+ cells was considerably increased in COVID-19 patients compared to HIV-1 patients (who have increased frequencies of these circulating precursors) (Fig 4E). High DNAM-1 molecule density is a hallmark of these cells, which are virtually absent in HD. Overall, in the whole cohort, a significant increase of “inflammatory” Lin-CD34+DNAM-1^bright^CXCR4^+^ NK cell precursors was observed compared to HD (27.5±24.07 vs. 3.01±2.93, p = 0.0013; Fig 4E), as well as compared to HIV-1 patients (27.5±24.07 vs. 9.74±11.01, p = 0.028; Fig 4F).

Taken together, these results support the view that there is an accelerated NK cell turnover in tissues and an increased recruitment of inflammatory precursors from the BM in COVID-19 patients, to a greater extent than previously observed elsewhere.

### Dysregulated NK cell immune landscape correlates with clinical disease outcomes

In view of the above findings, and of the rapidly divergent disease courses of the patients after admission, we next studied whether the NK cell parameters that were significantly perturbed could be prognostic of disease outcome, independent of subsequent treatment (which has been reported elsewhere [27].

**Fig 5:**
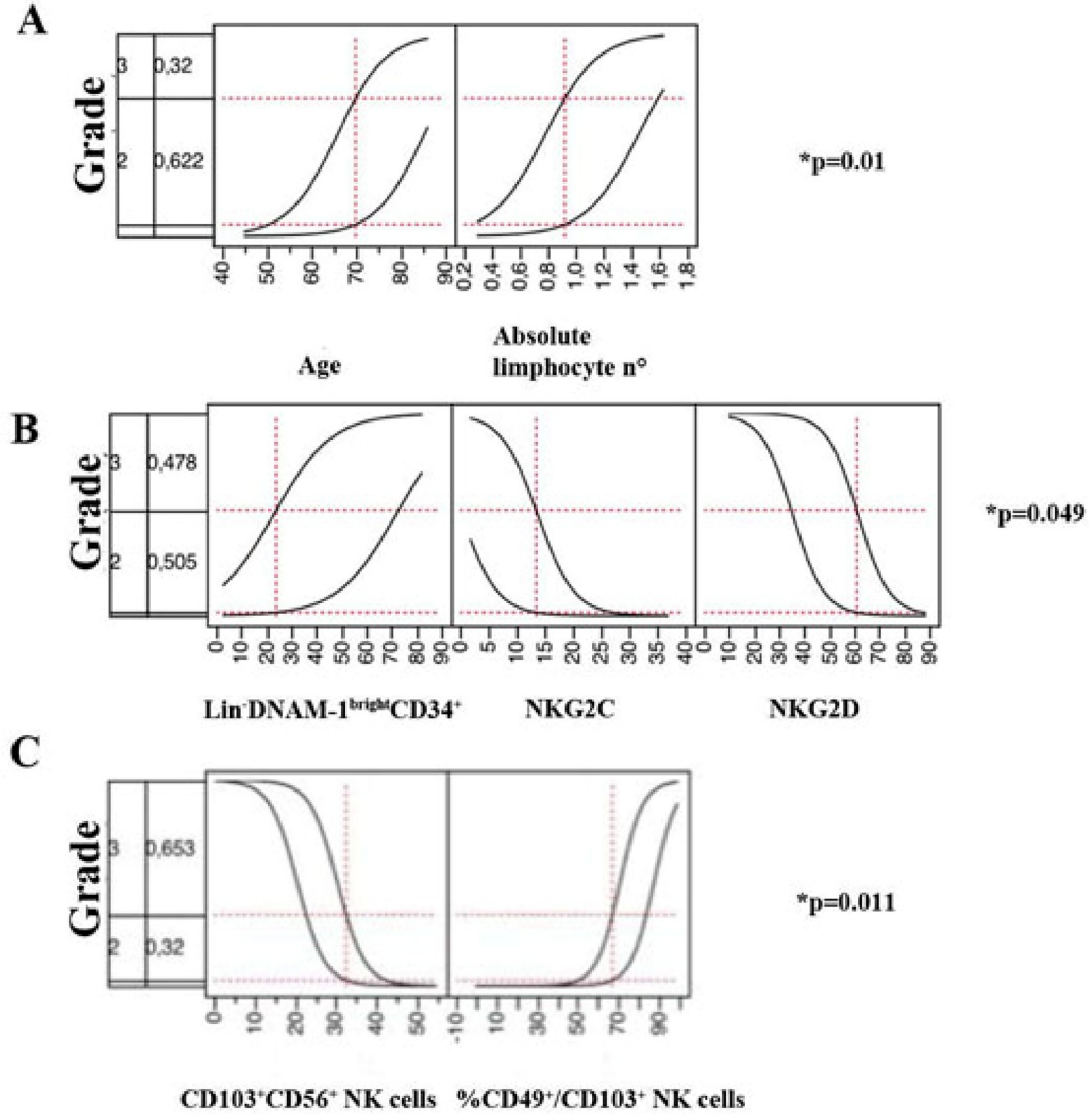
Ordinal logistic fit of variables with subsequent clinical trajectory according to modified WHO classification. **Panel A:** Logistic profiler for the disease course for age and lymphocyte n° at admission according to subsequent disease trajectory. Logistic fit of the model is indicated. The vertical dotted line for given *X* variables shows a representative *current value* (red number). Horizontal dotted lines show the *current predicted value* for the displayed values of the *X* variables. **Panel B:** Logistic profiler for disease course according to the proportion of CD34+DNAM^bright^CXCR4+ cells, the proportion of NKG2D+ and of NKG2C+ NK cells. Logistic fit of the model is indicated. The vertical dotted line for given *X* variables shows a representative *current value* (red number). Horizontal dotted lines show the *current predicted value* for the displayed values of the *X* variables. **Panel C:** Logistic profiler for disease course according to the proportion of CD103+ NK cells and of CD49+CD103+ NK cells. Logistic fit of the model is indicated. The vertical dotted line for given *X* variables shows a representative *current value* (red number). Horizontal dotted lines show the *current predicted value* for the displayed values of the *X* variables.

Ordinal logistic regression analysis was performed, evaluating NK cell parameters according to the highest disease severity grade reached during the entire hospital stay. As shown in Table 1 and in Fig 5A, age and leukocyte parameters were associated with disease severity (p = 0.01) with lower lymphocytes with increasing case severity scores. This was in line with previous reports and present knowledge[20], supporting the accuracy of patient categorization and leading us to proceed with NK cell parameter analysis.

By ordinal logistic regression, the proportions of NKG2D+ and NKG2C+ NK cells and of CD34+DNAM-1^bright^CXCR4+ stem cells (a proxy for peripheral NK cell turnover and central release) were correlated to disease course severity following the initial sampling (p = 0.0049).

The amount of tissue-trafficking NK cells (CD103+ and CD49d+CD103+) was also significantly associated with the disease course (p = 0.011).

Overall, therefore, NK cell trafficking and turnover, when evaluated at and around admission, are associated with disease course severity.

## Discussion

In this work, we show that COVID-19 patients at hospital admission display extensive activation of PB NK cells, accompanied by an imbalance and dysregulation of peripheral NK cell subsets, with a shift toward terminal differentiation and increased expression of inhibitory receptors. This is associated with exceedingly high tissue trafficking of otherwise tissue-resident NK cells, and to defective NK cell cytotoxic activity in the absence of perforin or triggering receptor expression.

The decreased proportions of CD16+/-CD56^bright^ and KIR-NKG2A+ NK cells observed here are in agreement with the increased peripheral maturation of NK cells following infection and virus replication, which has been previously reported in hematopoietic stem cell transplantation [37, 38]. Advancing age associates with an increase in the relative percentage of NK cells when comparing elderly and young individuals[39–41]. The rise in percentage of NK cells in the elderly is associated also with a progressive decrease in CD56^bright^ NK cells[41, 42]. The loss of cytotoxic NK function observed with age however [40] is associated predominantly not to numeric decrease in CD56^bright^ cells which are less cytotoxic, but rather to CD56^dim^ NK cells. The mean age of healthy controls in our groups was 54±14years as compared with 68.9±16.02 in the group of patients with most severe disease and 72±13 years overall. The difference in age may have accounted for part of the low CD56^bright^ cell proportions that was observed here, although the proportion of CD56^bright^ cells in the present study was considerably lower (1.9±1.4%) than the one reported for patients >60years of age (4%)[41].

COVID-19 patients exhibited an increased expression of inhibitory molecules, including both NKG2A and KIRs. This finding agrees with the previous report of increased isolated NKG2A expression in T cells and NK cells [22] and of functionally relevant increases of NKG2A and of KIR2DL1/S1 and PD-1[43]. These observations add important details in this respect. Indeed, the increased NKG2A expression was represented by high molecule density rather than by increased NKG2A+ cell frequency. Overall, the significant reduction of NKG2A+KIR- immature NK cells, decrease of CD56^bright^CD16+/- cells and increase of “memory”-like CD85j+CD57+KIR+ NK cells all contribute to the interpretation of a peripheral skewing of circulating NK cells towards accelerated maturation. In this context, the increased density of NKG2A molecules occurs on “maturing” NKG2A+KIR+CD56^dim^CD16+ cells. The concomitantly increased KIR molecule density observed here contributes to a widely increased HLA class I and HLA-E-mediated inhibitory control on NK cell activation signaling and is in line with previous reports suggesting that NK cells are in a dysfunctional state in COVID-19 patients[22, 43].

The possibility that the skewing toward more mature NK cell subsets is associated with increased peripheral NK cell turnover was confirmed here by the observation of increased levels of “emergency” CD34+DNAM-1^bright^CXCR4+ precursors that rapidly generated mature NK cells in vitro [29]. These precursors have been previously observed during chronic HIV-1 and HCV infections, and are known to exit the bone marrow during intense inflammation, generating rapidly functional NK cell progenies with a mature KIR+ phenotype already in vitro [29]. Their increase in COVID-19 patients was considerably higher than in HIV patients, indicating a surprisingly high level of inflammation and peripheral turnover. This interpretation is also supported by the high levels of NK cell activation in COVID-19 patients. Indeed, HLA-DR expression by NK cells has been reported as a sign of intense chronic activation during untreated and treated HIV-1 infections [14, 15], and is accompanied in this condition by the inflammatory precursor exit from the bone marrow following inflammation and peripheral NK cell turnover [29]. The mechanism(s) underlying this condition in COVID-19, however, appear to be different from those present in other viral infections. Indeed, the reduced NK cell function observed in SARS-CoV2-infected patients did not depend on perforin for activating NCR expression, as previously reported for HIV-1 infections [14, 15, 44], since NK cells have decreased triggered cytotoxicity, decreased total cytolytic potential, but normal NCR expression, as well as normal perforin content. This decreased NK cell function is particularly surprising in view of their mature phenotype. Virus-infected cell recognition by NK cells is not pathogen-specific in the vast majority of cases, but rather depends on recognition of cell ligands to activating receptors that are induced by virus infection (e.g. PVR, Nectin-2, MIC-a, MIC-b, ULBPs, B7-H6 etc). Accordingly, we here show that NK cell function in COVID-19 patients is strongly reduced in a redirected killing assay that mimicks triggering of activating receptors upon encounter of autologous virus-infected cells without the interference of inhibitory signals (e.g. those mediated by NKG2A or KIRs). Thus, it can be assumed that virus-inhibiting activity of circulating NK cells would be expected to be similarly abolished, if this could not be verified since we did not have access to biosafety SARS-CoV-2 manipulation for cytotoxicity assay. Nevertheless, future work will need to address these models to confirm the observation and to verify whether other endemic coronavirus (HCoV-NL63, -229E, -OC43 or -HKU1) could serve as a proxy for anti-SARS-CoV-2 NK cell function.

Mechanism(s) other than triggering receptor function are probably responsible for this functional impairment, and could be associated with increased (KIR- or NKG2A-mediated) inhibition, or to functional NK cell attitude, considering the significant circulation of tissue-resident cells.

Indeed, the reciprocal HLA-DR and CD69 expression in NK cells observed here is unprecedented, and, together with the significant circulation of CD69+CD49d+CD103+ NK cells, signals an extensive trafficking derangement during symptomatic SARS-CoV-2 infection. CD69, CD103, CD49a, and CXCR6 promote tissue retention, and are used to describe tissue-resident NK cells (trNK) in general [34, 45]. More recently, it has been established that healthy lungs contain a small subset of CD69+CD49a+CD103+ trNK, distinct from the peripheral NK cells [36, 47], that are functional, degranulate, and show an increased release of granzyme and IFN-γ upon influenza A virus infection in vitro [46]. In this context, the present findings in COVID-19 patients indicate that during early phases of the disease, there is an increased trafficking of trNK cells. The reciprocal expression of CD69 and HLA-DR, as well as the increased circulation of “inflammatory” CD34+ precursors (CD34+DNAM-1^bright^CXCR4+), could be interpreted to reflect flow into (lung) tissues of not yet activated NK cells following SARS-CoV-2 replication and inflammation. It should be noted that in the present work we only defined trafficking, but have no clue on the origin of circulating tissue-resident NK cells. The trafficking may be due to trNK cells released from other tissues in a finalistic attempt to seed the lungs. Alternatively, since these cells have a phenotype quite similar to the recently described lung-resident NK cells, one cannot rule out that they may be released from damaged lung tissue.

A limit to the present data interpretation is represented by the absence of a parallel control of patients with primary community-acquired viral pneumonias (e.g. Influenza A, metapneumovirus, parainfluenza virus) on their early hospitalization, to verify whether and how the present observations apply only to SARS-CoV-2 and not to other viruses. The exclusion of concurrent viral pneumonia circulation during the SARS-CoV-2 epidemic prevented from parallel evaluation in this regard[28]. Although to our knowledge this is the first time that these alterations in NK cell subsets and function are reported during primary viral pneumonia, future work will need to verify uniqueness of this phenomenon.

With regard to an association between the NK cell phenotype and disease trajectories, disease severity in this cohort was directly associated with the frequency of circulating CD103+CD49d+ cells and of CD34+DNAM-1^bright^CXCR4+ precursors, while increased proportions of NK cells expressing activating receptors (NKG2D+, NKG2C+) were protective against the worst outcome (characterized by need for mechanical ventilation). In line with these findings, an association between NK cell phenotype/function and the disease course has been described during other viral infections. Indeed, NK cell surface molecule expression and transcriptomic analysis predicts the success or failure of HCV treatment with pegylated IFN2a-ribavirin [47, 48]. Also, NCR inducibility is associated with a benign disease course in HIV infections [49, 50]. This observation thus further supports the possibility of an association between a fine NK cell mechanism and some aspects of clinical variability. This interpretation of the results is supported the recent description of COVID-19’s association with the peripheral immune phenotype [51], which, however, only marginally investigated NK cell parameters. In addition, memory-like (NKG2C+) NK cell phenotype depends on CMV serostatus[52] which is positive in up to 90%of those aged 75-90years as is the case in these COVID-19 patients[53]. Accordingly, the present finding among a cohort of patients with a very high likelihood of increased CMV seropositivity that those with higher frequency of NKG2C+ cells have better disease outcomes is in line with current knowledge that improved NK cell function in elderly patients associates with less infection-related disease[40].

Thus, the present fine immune landscape analysis of NK cell parameters and their precursors upstream of described adaptive T and B cell responses is consistent with and elaborates on this view [51]. It should be noted, however, that this study has several limitations, including the patient sample size, the lack of a validating set of patients, and the lack of a modeling comprehensive of treatment effects. Therefore, this analysis confirms the general observation that there is an increased peripheral NK cell turnover and trafficking that indicates severe COVID-19, but additional work is needed to verify whether and how these parameters could be of clinical use.

The present work was not set to provide mechanistic explanations underlying the generation of the NK cell landscape with dysfunctional NK cells described here and elsewhere[22, 43], associated with the finding of abundant trNK cells in PBMC that derive from tissues that may include the lung or sites of ongoing virus replication. Increased circulation of inflammatory precursors that generate mature and functional NK cell progenies[29, 54] signals the utterly elevated tissue turnover of NK cells and answers one of the relevant questions with regard to NK cells in COVID-19. A possible picture emerges of increased influx of functional NK cells into inflamed tissues, increased expression of inhibitory molecules contributing to NK cell dysfunction and trNK cell efflux from inflamed or injured tissues. Future work is required to clarify the underlying mechanisms.

In conclusion, we identify a unique immune signature of circulating NK cells in symptomatic SARS-CoV-2-infected patients, characterized by intense NK cell activation, functional derangement, and turnover, associated with the circulation of putative tissue resident CD56+CD16+CD69+CD103+CD49d+ NK cells. Since NK cells make up one-fifth of lung parenchymal lymphocytes, their manipulation may well provide robust and broad innate immune responses to SARS-CoV-2 infection. Overall, these insights into the mechanism of early derangements of innate responses suggest that further exploration of these NK subsets is required to understand how local mucosal immunity could be manipulated to support SARS-CoV-2 vaccine development.
